# Postural control of sway dynamics on an unstable surface reduces similarity in activation patterns of synergistic lower leg muscles

**DOI:** 10.3389/fspor.2025.1545813

**Published:** 2025-03-12

**Authors:** Lida Mademli, Maria-Elissavet Nikolaidou, Sebastian Bohm, Adamantios Arampatzis

**Affiliations:** ^1^ Laboratory of Adapted Physical Education, School of Physical Education and Sport Science (Serres), Faculty of Physical Education and Sport Sciences, Aristotle University of Thessaloniki, Thessaloniki, Greece; ^2^ Sports Biomechanics Laboratory, School of Physical Education and Sport Science, Faculty of Physical Education and Sport Science, National and Kapodistrian University of Athens, Athens, Greece; ^3^ Department of Training and Movement Sciences, Humboldt-Universität zu Berlin, Berlin, Germany; ^4^ Berlin School of Movement Science, Berlin, Germany

**Keywords:** triceps surae muscle, quadriceps femoris muscle, balance control, diversity of activation pattens, flexibility of neuromotor control

## Abstract

**Introduction:**

Diversity of activation patterns within synergistic muscles can be important for stability control in challenging conditions. This study investigates the similarity of activation patterns within the triceps surae and quadriceps femoris muscles and the effects of unstable surface during a visually guided postural task.

**Methods:**

Eighteen healthy adults performed a visually guided anteroposterior tracking task on both stable and unstable surfaces. Electromyographic activity of triceps surae (gastrocnemius medialis, gastrocnemius lateralis, soleus) and quadriceps femoris (vastus medialis, vastus lateralis, rectus femoris) was recorded at 1,000 Hz. Cosine similarity (CS) between muscle pairs within each muscle group was calculated to assess the similarity of activation patterns of synergistic muscles for stable and unstable conditions. To compare the CS of the muscle pairs, a linear mixed model was used. For all tests the level of significance was set to *α* = 0.05.

**Results:**

Across all surface conditions, CS values within the triceps surae muscles were lower than those of the quadriceps (*p* < 0.001), indicating a greater diversity in activation patterns of the distal muscles. The unstable surface reduced CS values for both muscle groups (*p* = 0.021). No significant interaction was observed between muscle pair and surface condition (*p* = 0.833).

**Discussion:**

The reduced similarity of activation patterns within the synergistic triceps surae and quadriceps femoris muscles on the soft surface indicates an increased flexibility of neuromotor control for the unstable condition. The lower similarity within the synergistic triceps surae muscles suggests a higher diversity of activation patterns compared to the quadriceps femoris muscles, which may increase the flexibility of neuromotor control to meet specific joint stabilization challenges during the studied tracking task.

## 1 Introduction

Successful movement requires efficient control of the human musculoskeletal system by the central nervous system. Given the large number of degrees of freedom within the musculoskeletal system, this control process is highly complex and not yet fully understood. To address this complexity, it has been proposed that muscles are controlled through muscle modules, whereby a single neural command activates a group of muscles, thereby reducing the dimensionality of movement control (1, 2). The modular organization of lower leg muscle activation affects the efficiency, robustness and mechanical loading of the joints (3–5) and is therefore important in everyday tasks such as standing balance (6, 7), walking and running (8–11), landing (12, 13) and perturbed locomotion (14–16). During challenging conditions on unstable surfaces, such as locomotion, transitioning from double to single-leg stance, landing or lunging on unstable surfaces, the human neuromotor system compensates and enhances its robustness by modulating muscle synergies, i.e., by widening the time dependent components of muscle synergies (7, 12, 17, 18). Furthermore, muscle coactivation around knee and ankle joint increases, when performing balance or postural tasks on unstable conditions (12, 18, 19) with the increase being more pronounced around the ankle joint (12, 18). In the above mentioned studies, the triceps surae muscles were reported to be involved in the same module as synergistic muscles. The same is also true for the synergistic quadriceps muscles (3, 13, 17). However, this synergistic behavior seems to be more complicated, since it has been suggested that not all anatomically deﬁned synergist muscles share a strong common neural drive (20–22). It has been suggested that an independent drive to certain muscles within the same muscle group enables more flexible control (21), allowing for a redistribution of neural drive across muscles to cope with challenging neuromotor task conditions (22).

For instance, while a strong common drive between muscles such as vastus lateralis and vastus medialis might be necessary to protect the knee joint from excessive internal contact stresses (23, 24), other synergistic muscles, such as the two gastrocnemii, might require a more ﬂexible control strategy to comply with secondary goals, e.g., ankle stabilization (20, 21). These differences between synergistic muscles suggest possible diversity in the activation patterns, which may affect the force-sharing among the synergistic muscles. Lai et al. (25) and Hamard et al. (26) reported a diversity in electromyographic (EMG) activity among the synergistic triceps surae muscles during walking and running. In situations where the application of forces is required in multiple directions, such as around joints responsible for postural control like the ankle, greater flexibility in muscle control may be required to manage varying external demands. A diversity of activation patterns among synergistic muscles may improve task performance. Under conditions of increased postural demands, the need for more flexible motor control becomes greater, and a wide range of possible activation strategies among synergistic muscles may increase the versatility for effective control and regulation of body stability. However, to the best of our knowledge, no study has yet examined the similarity of activation patterns within different muscles groups in both stable and unstable balance conditions.

The primary aim of this study was to determine (1) whether the similarity of activation patterns within the distal triceps surae muscles differs from that within the more proximal quadriceps femoris muscles during a visually guided postural task, and (2) whether the similarity of activation patterns within synergistic muscles is altered under unstable conditions. To create an unstable and thus a more challenging condition for the neuromotor system, we introduced surface-related perturbations by having participants standing on a foam surface while performing a visually guided postural task. In previous research, we found that these surface-related perturbations had a significant impact on the local dynamic stability, with greater short-term maximum Lyapunov exponents (sMLE) values for the whole-body kinematics (from head to foot), implying that tracking a visual moving target on an unstable surface is a more demanding and more challenging task than doing so on a stable surface (19).

We studied the activation patterns of synergistic muscles from two different muscle groups—the ticeps surae and quadriceps femoris—as previous literature indicates that they differ in the level of common neural input shared between their muscle heads. To quantify the similarity of the activation patterns between the different muscles within the same muscle groups, we used the cosine similarity (27, 28). First, based on previous findings of increased shared neural input between the vastus lateralis (VL) and vastus medialis (VM) muscles compared to the gastrocnemius lateralis (GL) and gastrocnemius medialis (GM) muscles (22), we hypothesized that during a visually guided postural task, the similarity of the activation pattern within the quadriceps femoris muscles would be greater than that within the triceps surae muscles. Second, based on reports that unstable surfaces increase body instability during visually guided postural tasks (19, 29), we hypothesized a decrease in the similarity of activation patterns within the synergistic triceps surae and quadriceps femoris muscles in the unstable condition.

## 2 Methods

### 2.1 Participants

Twenty healthy young adults (age 32 ± 5 years, mean ± standard deviation) with no history of neuromuscular or musculoskeletal disorders participated in the study (12 male and 8 female, body height 175 ± 7 cm, body mass 69 ± 12 kg). From two participants not all data (EMG activity, ground reaction forces) were accurately collected for all examined trials, thus the data from 18 adults were used in the analysis (11 males and 7 females, body height 175 ± 8 cm, body mass 72 ± 11 kg, age 32 ± 6 years). All participants were informed about the experimental protocol and gave their informed consent before their inclusion to the study. The experiment was performed with the approval of the institution's ethics committee (HU-KSBF-EK_2018_0013, Humboldt-Universität zu Berlin) and in accordance with the Declaration of Helsinki.

### 2.2 Task and apparatus

**Figure 1:**
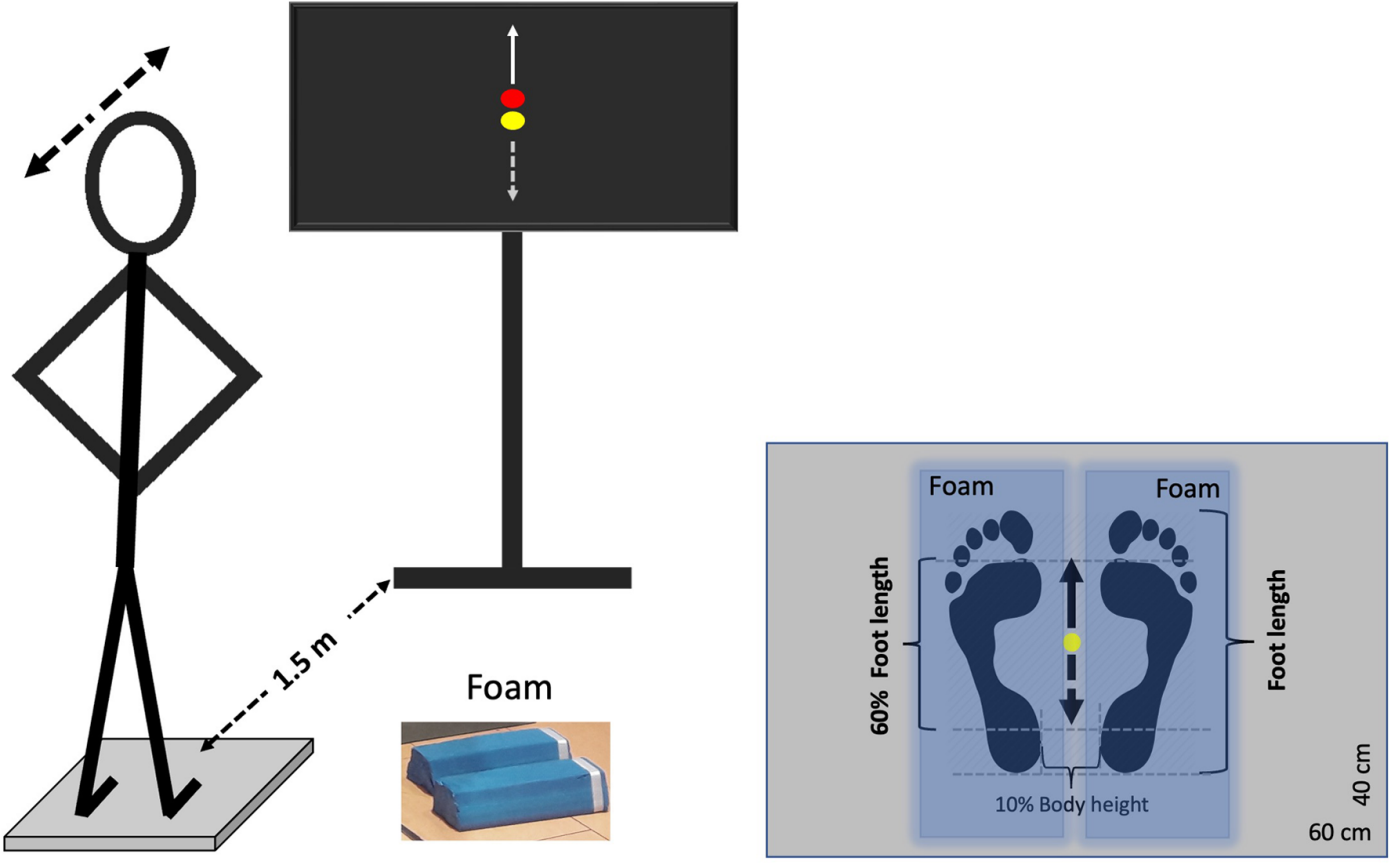
Experimental set up. Participants tracked a visual target (red dot) moving vertically using a yellow dot, which was controlled by their center of pressure (CoP) movement in the anteroposterior direction. They maintained their feet on the ground and leaned their body forward and backward. An anterior CoP shift corresponded to an upward movement of the yellow dot (solid arrow), while a posterior shift corresponded to a downward movement (dashed arrow). The target moved sinusoidally at 0.25 Hz, with an amplitude normalized to 60% of the participant's foot length. The task was performed on both a stable (rigid) surface and an unstable (foam) surface. Blue-shaded areas indicate the position of the foam pads for the unstable trial. Each tracking task lasted 120 s.

Participants had to perform a postural tracking task by shifting their weight anteroposteriorly during standing on a force plate (40 × 60 cm, AMTI BP400600-2000, Advanced Mechanical Technology, Inc., Watertown, MA, USA) under two different conditions: (a) on a stable surface (floor) and (b) on an unstable surface (foam). The foam consisted of two balance beams (Sport-Thieme Balance beam EVA foam, with the dimensions 38 × 16.5 × 5.8 cm, Sport-Thieme Germany), which were oriented in the foot's anterior to posterior plane. The tracking task involved following a visual target, which was displayed on a TV screen (47″, HD LG) as a red dot and moved vertically in the center of the screen. In addition to the target, the TV screen displayed a yellow dot representing the participant's anteroposterior center of pressure (CoP) component, providing real-time feedback of the participant's position on the force plate. Both the target and CoP position feedback were synchronously shown on the TV monitor by means of a custom-built software, developed in MATLAB R2014 (Math Works Inc., USA), with 50 Hz refresh rate. Participants were instructed to follow the red dot's movement by adjusting the yellow dot through forward and backward weight shifts, while keeping their knees and hips straight. Specifically, when the red dot moved upward, participants had to shift their weight anteriorly, and when it moved downward, they had to shift their weight posteriorly. Each participant performed one trial per condition in a randomized order to prevent sequence effects, with each trial lasting 120 s. Prior to each trial, there was a familiarization period lasting maximum 30 s. During the trial, participants stood barefoot on the force plate with their feet at the center of the platform, maintaining an inter-malleolar distance equal to 10% of their body height. They held their arms akimbo to ensure stability and eliminate any influence of arm movements on balance and task performance. The experimental set up is depicted in Figure 1.

The target's motion was periodic sinusoidal with a single frequency (f) set at 0.25 Hz, which was generated in MATLAB R2014b using the sine function [sine(t)=sin(2πi×f×t)]. This frequency was selected because it is the dominant frequency of natural, self-paced voluntary sway (30, 31). The maximum (peak to peak) amplitude of target motion was adjusted for each participant to 60% of his/her foot length, which corresponded to an average amplitude of 15.08 ± 1.09 cm. This amplitude was chosen based on pilot tests showing that 60% of foot length is suitable for swaying on both stable and unstable surfaces, as it effectively increases instability on the unstable surface compared to the rigid one, consistent with findings from earlier studies (7, 17).

The participants' postural behaviour was recorded using the force platform at 1,000 Hz. The EMG activity of soleus (SOL), GM, GL, rectus femoris (RF), VL and VM was captured at a sampling frequency of 1,000 Hz using a wireless EMG system (myon m320, myon AG, Schwarzenberg, Switzerland). The wireless electrodes were placed at the right lower limb, on the belly of each muscle, with a 2 cm intra-electrode distance according to SENIAM recommendations. All data (force and EMG signals) were simultaneously recorded by a motion capture system (Vicon, Oxford, U.K.). An interface was created in MATLAB R2014b for triggering and synchronizing all devices with a single pulse.

### 2.3 EMG processing

EMG activity was processed and analysed in MATLAB R2023b. First, for each trial and participant, the data of the first cycle of the target motion were excluded for any further analysis, since most of the participants were not able to follow the target accurately during this first cycle. The raw EMG signals were high-pass filtered at 20 Hz using a 4th-order Butterworth zero-lag filter to remove movement artifacts and low-frequency noise. The signals were then full-wave rectified and smoothed with a low-pass filter at 5 Hz using a 4th order Butterworth zero-lag filter to extract the envelope of the EMG activity. The filtering was applied sequentially, with high-pass filtering preceding rectification and low-pass smoothing.

To normalise the EMG signals, a single average reference value was calculated for each participant and each muscle from the filtered and rectified EMG signal from both the stable and unstable conditions. Specifically, for each muscle the mean of the EMG signal was calculated separately for the stable and unstable condition over the entire duration (excluding the first cycle). The average of these two mean values was then used as a constant reference to normalize the EMG signals for each corresponding muscle and participant for both conditions. To account for baseline muscle activation, the minimum value of the normalized EMG signal of the two conditions (stable and unstable) was identified for each muscle. The lower of these two values was then subtracted from the normalized EMG to remove the baseline activation for each muscle and each participant in both conditions. Henceforth, any reference to the EMG signal denotes the EMG signal that has been filtered, normalized, and adjusted for baseline activation.

The cosine similarity (CS) between the EMG signals of different synergistic muscle pairs was calculated in MATLAB R2023b, to assess the similarity between the EMG signals of the examined muscle groups (27, 28) under both stable and unstable conditions. The CS between each pair of EMG signals was calculated using the dot product of the two signals divided by the product of their norms. This was done separately for the stable and unstable conditions using the following equation:

$$CS_{\mathrm{EMGi}}=\frac{\mathrm{dot}\;(\mathrm{EM}G_{x},\;\mathrm{EM}G_{y})}{∥\mathrm{EM}G_{x}∥\;\;\;\times \;\;\;∥\mathrm{EM}G_{y}∥}$$

where $CS_{\mathrm{EMGi}}$ is the cosine similarity value for a given muscle pair and condition, $\mathrm{EM}G_{x}$ and $\mathrm{EM}G_{y}$ represent the EMG signals of the two different muscles. Note that cosine similarity values can range from 1, indicating a perfect match in pattern, to −1, indicating a perfect inverse pattern.

The CS was calculated at the stable and unstable conditions for the EMG signals of the following synergistic muscle pairs: GM-GL, GM-SOL and GL-SOL for the triceps surae muscle, and RF-VL, RF-VM and VM-VL for the quadriceps femoris. Furthermore, the CS between the target signal and CoP was also calculated to evaluate the participants' accuracy in tracking of the moving visual target under both conditions. This was accordingly done by computing the dot product of the anteroposterior coordinates of the CoP during each tracking task with the sinusoidal signal representing the target's motion and then dividing the outcome by the product of their norms.

### 2.4 Statistical analysis

All statistical analyses were performed using IBM SPSS statistics (version 29, Armonk, NY:IBM Corp). The normality of the CS data was confirmed with the Shapiro–Wilk test. The values of CS between the CoP and Target, representing the accuracy of CoP-Target motion coupling, were compared between the stable and unstable conditions using a dependent samples *t*-test. A linear mixed model was used to compare the CS of the EMG signals between the examined muscle pairs, groups, and surface conditions. The model included one random factor (participants) to account for the repeated measures within individuals, and three fixed factors: condition (stable vs. unstable surface), muscle pair (GM-GL, GM-SOL, GL-SOL, VL-VM, VL-RF, VM-RF), and muscle group (triceps surae vs. quadriceps femoris). *post hoc* pairwise comparisons using dependent samples *t*-test with the Sidak correction to adjust for multiple comparisons were conducted to explore significant main effects and interactions between the three fixed factors. The normality of the residuals for the linear mixed model was assessed using the Shapiro–Wilk test and Q-Q plots. While the majority of the data were normally distributed, deviations were observed in the residuals for CS of two muscle pairs (RF-VM in the stable condition and VM-VL in the unstable condition). These deviations were considered acceptable given the robustness of the linear mixed model to minor violations of normality (32). Further, residual diagnostics, including Q-Q plots and scatter plots of residuals vs. predicted values, were examined to ensure that the assumptions of normality, homoscedasticity, and linearity were reasonably met. For all tests the level of significance was set to *α* = 0.05.

## 3 Results

**Figure 2:**
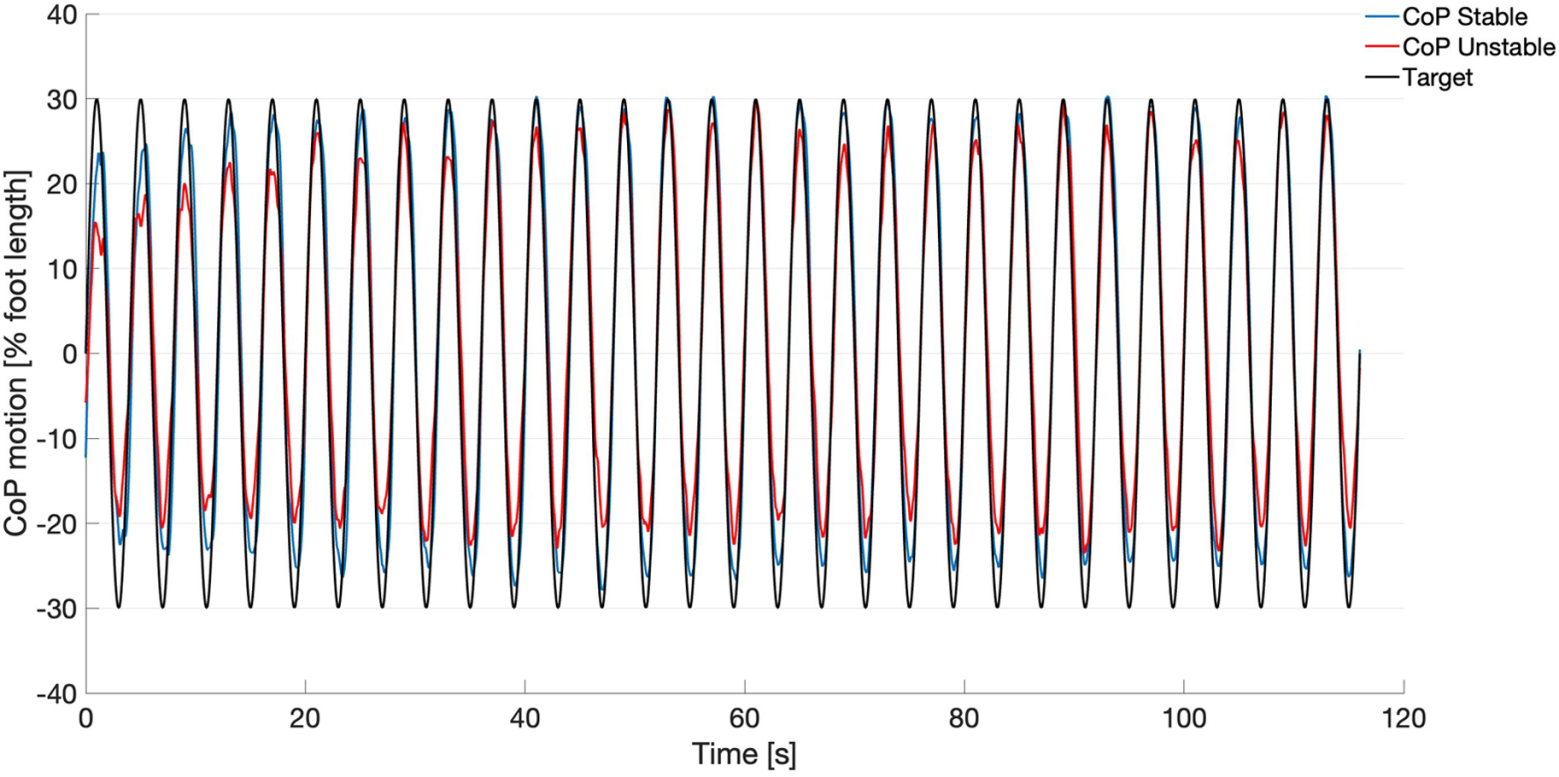
Target signal (black) and mean curves of the center of pressure (CoP) motion from all participants (*n* = 18) during the tracking task on the stable surface (blue) and on the unstable surface (red).

In Figure 2, the target signal and the group averaged CoP displacement on both stable and unstable surfaces are depicted. The CS values between CoP and Target for all participants ranged from 0.79–0.95 for the stable condition and from 0.66–0.93 for the unstable condition. The pairwise comparison revealed significantly greater values for the stable condition (0.91 ± 0.04) compared to the unstable condition (0.84 ± 0.07, *t*(14) = 3.917, *p* = 0.002).

**Figure 3:**
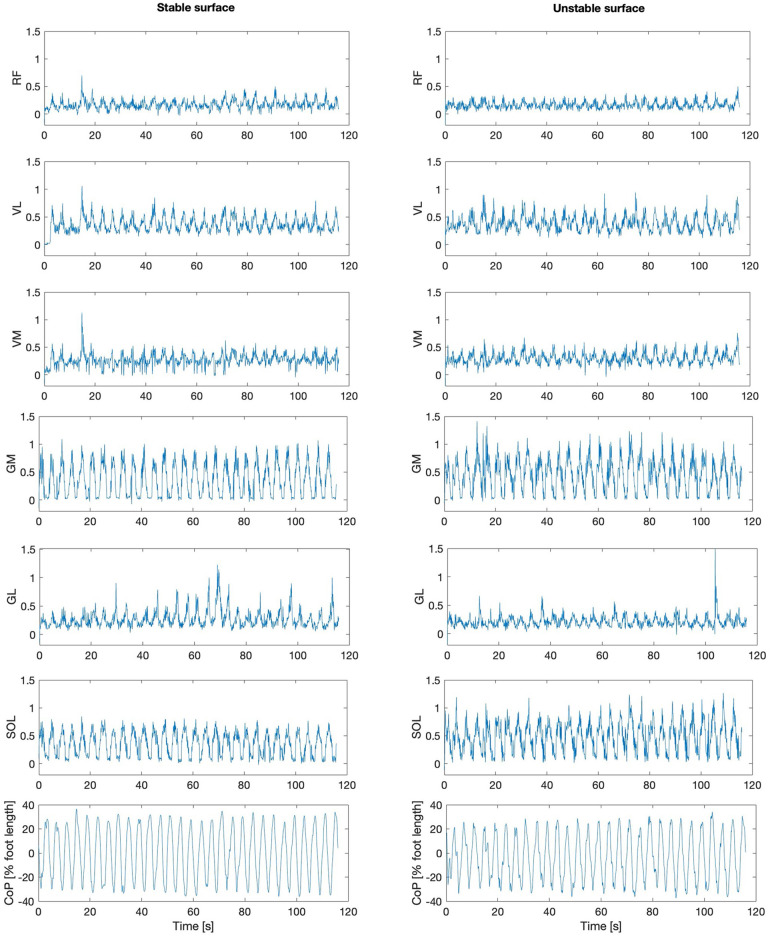
The center of pressure motion (CoP) and the processed electromyographic activity [mV] of all examined muscles: rectus femoris (RF), vastus lateralis (VL), vastus medialis (VM), gastrocnemius medialis (GM), gastrocnemius lateralis (GL), and soleus (SOL), during the postural tracking task on the stable (left) and on the unstable surface (right) are depicted exemplarily for one participant.

**Figure 4:**
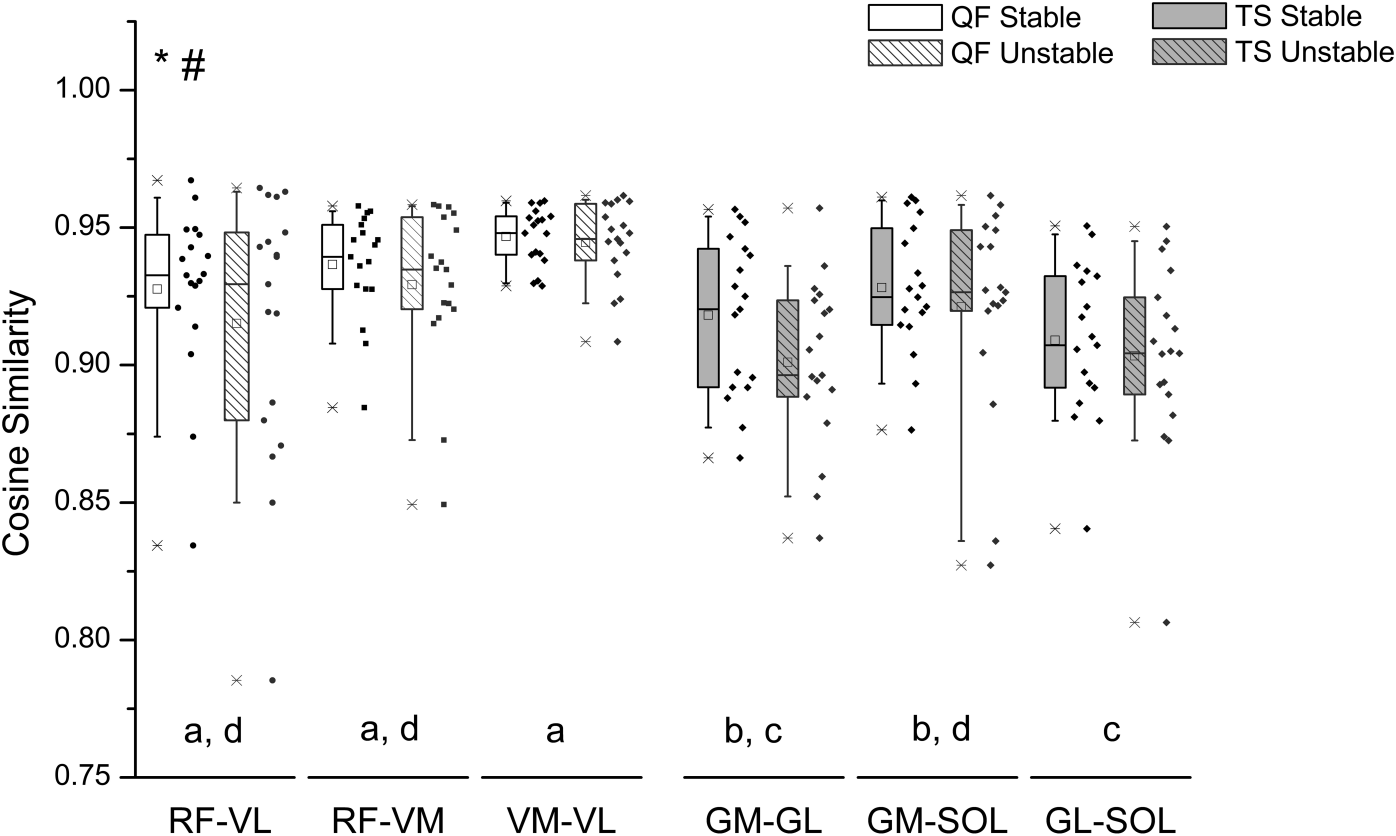
Box plot and individual data points (dots) of the cosine similarity values between the EMG activity of quadriceps femoris (QF) and triceps surae (TS) muscle pairs during postural tracking on the stable (unfilled bars) and on the unstable surface (striped bars). The center line of each box denotes the median value (50th percentile), while the box spans the interquartile range (25th to 75th percentiles). The whiskers mark the 10th and 90th percentiles. RF, rectus femoris; VL, vastus lateralis; VM, vastus medialis; GM, gastrocnemius medialis; GL, gastrocnemius lateralis; SOL, soleus. *Statistically significant differences between QF and TS muscles (*p* = 0.001). ^#^Statistically significant differences between stable and unstable condition (*p* = 0.021). Muscle pairs sharing the same letter do not differ significantly (*p* > 0.05, *post hoc* analysis).

The EMG activity of the examined muscles for one participant is indicatively depicted in Figure 3 for both stable and unstable condition. Overall, there was a significant surface effect on the CS values between the EMG signal of all synergistic muscle pairs [*F*(1, 187) = 5.425, *p* = 0.021], with lower values for the unstable surface (Figure 4), indicating less synchronized muscle activation patterns on the unstable surface independent of muscle group.

For all surface conditions the quadriceps femoris muscle group demonstrated greater CS values than the triceps surae muscle group [*F*(1, 187) = 56.529, *p* < 0.001] (Figure 4). When comparing the CS between muscle pairs, significant differences were found, independent of surface condition [*F*(4, 187) = 4.582, *p* = 0.001]. *post hoc* pairwise comparisons revealed significant differences in CS between the examined muscle pairs (Figure 4). Specifically, the CS of muscle pairs GM-GL and GL-SOL was significantly lower than that of all quadriceps femoris muscle pairs (i.e., RF-VL *p* < 0.01, RF-VM *p* = 0.01, VM-VL *p* < 0.01). The CS of muscle group GM-SOL was greater compared to GL-SOL (*p* = 0.019) and lower compared to VM-VL (*p* = 0.004). Within the quadriceps femoris muscle group, no significant differences in CS values were found between muscle pairs (RF-VL vs. RF-VM: *p* = 1.000, RF-VL vs. VM-VL: *p* = 0.753, RF-VM vs. VM-VL: *p* = 0.227). There was no significant interaction between muscle pair and surface condition [*F*(4, 187) = 0.366, *p* = 0.833].

## 4 Discussion

In the present study, the CSs of the synergistic muscle pairs were higher in the quadriceps femoris compared to the triceps surae muscles, indicating superior similarity in the activation patterns of the more proximal muscles during the tracking task studied, confirming our first hypothesis. Greater synchronization between the quadriceps femoris compared to the triceps surae muscles was also reported by Rossato et al. (22) during isometric contractions. The authors argued (22) that more independent synaptic input within synergistic muscles may provide more flexible neuromotor control to meet specific joint stabilization challenges. The human triceps surae muscles are pennate with a substantial in series elasticity due to the long Achilles tendon. Pennate muscles with substantial in series elasticity are particularly sensitive to external perturbations and capable of flexible force generation for body stability (33, 34). Although the three synergistic triceps surae muscles are pennate with high in series elasticity, they have important differences in terms of function and geometry. The biarticular GM and GL muscles have variable frontal moment arms, resulting in different functional outcomes across the frontal plane range of motion (35). Furthermore, their activation can be altered during balancing tasks due to the different mechanical advantages of the GM and GL muscles (20). The ankle joint plays a key role in human body stability during balance tasks (36, 37). Consequently, during the studied tracking task, a greater diversity of activation patterns within the triceps surae muscles may increase the flexibility of neuromotor control to manage secondary goals, such as producing opposing ankle moments in the frontal plane to maintain mediolateral body stability.

The quadriceps muscles are mainly involved in stabilizing the knee joint and may not be as functionally relevant as the triceps surae muscles for the chosen tracking task. During the examined postural task, quadriceps femoris muscles function primarily as damping elements to control the movement and secondarily as active components to generate force in the direction of the movement (29). It can be argued that the need for increased functional flexibility between the synergistic VL, VM and RF muscles is not as high as for the three triceps surae muscles, resulting in greater similarity in their activation patterns. The quadriceps muscles are also crucial in preventing patellofemoral pain as they control the internal stresses of the joint (23, 24). High synchronization between VM and VL minimizes mediolateral patellar force by balancing opposing forces on the patella (24). Thus, a higher similarity of the activation patterns within the quadriceps femoris muscles may represent a neural drive mechanism to protect knee joint from elevated patellofemoral joint contact stresses, which ultimately leads to pain.

We modified the surface with soft beams to create external mechanical perturbations for the participants during the tracking task. Although, there was a deterioration in tracking performance in the unstable condition, as reflected by lower CS values between target and CoP motion, all participants were still able to track the target despite the perturbations. This implies that the neuromotor system was capable of compensating for these perturbations, indicating a robust motor control. We also found a decrease in CS in both the triceps surae and quadriceps femoris muscles in the unstable condition, confirming our second hypothesis. This finding indicates adjustments in neuromotor control within the investigated synergistic muscles. The lower CS values within the triceps surae and quadriceps femoris muscles suggest increased diversity of individual muscle activation patterns and a strategy for more flexible, task-specific control of muscle activity on the unstable surface. The reduced similarity in the activation patterns of synergistic muscles may allow for greater and thus finer adjustments in force and stabilization, which are critical in unstable conditions.

Between the triceps surae muscle pairs we found different similarities in their activation patterns. The CS between GL-SOL were lower than GM-SOL, suggesting different functional contributions of the gastrocnemii during the tracking task. In stable and unstable conditions, the primary role of triceps surae muscles is to counteract forward body motion, while dorsiflexors regulate backward sway (19, 38). From the plantarflexors SOL plays a key role in postural control, acting as an antigravity muscle (39), given its larger physiological cross-sectional area (40) and predominance of type I muscle fibers (41). This stabilizing function is essential on both stable and unstable surfaces, providing sustained postural control when torque demands increase (19). The two gastrocnemii may produce different ankle moments in the frontal plane (35), and thus different patterns of activation during the tracking task may contribute to stability challenges in the mediolateral direction. It should be mentioned that in the triceps surae two biarticular muscles are involved and one in the quadriceps femoris muscle. Biarticular muscles generate moments in two joints simultaneously and can transfer energy between the two joints (42, 43). Biarticular muscles such as the gastrocnemii and rectus femoris showed greater changes in EMG-activity patterns compared to monoarticular muscles during step-down perturbations (44) and surprise loading (45) and may have a greater effect on similarity. When standing on a foam surface, the demand of postural stabilization and torque generation around the ankle joint increases due to the mechanical disturbances from surface compliance in both mediolateral and anteroposterior directions (46). Previous studies confirmed an increased EMG activity of muscles controlling anteroposterior motion, i.e., SOL and tibialis anterior, to counterbalance the demand for increased torque around the ankle joint due to the soft foam surface (19, 29). However, muscles primarily responsible for eversion, like peroneus longus, do not show this effect (29), possibly due to the anteroposterior emphasis of the task, which may allow mediolateral perturbations to be managed through control mechanisms other than increased activation, such as altered synchronization between GM-SOL and GL-SOL.

Our findings are related to the tasks investigated and the participants recruited for this study. It is possible that in more challenging tasks, such as tracking an unpredictably moving target or relying on acoustic rather than visual guidance, the effect of an unstable surface on the similarity of activation patterns within synergistic muscles could differ. Furthermore, individual skill level, training experience, and pathology may influence the degree of similarity between synergistic muscles. Nevertheless, we are confident that our main findings—reduced similarity in the triceps surae compared to the quadriceps muscles and a decrease in similarity due to surface instability—are valid. A further limitation of this study is that only a single-frequency visual target movement (0.25 Hz) was used, meaning that changes in muscle activation pattern similarity at different movement speeds were not explored. However, a previous study found that when performing the same task at a slower target speed (0.125 Hz), the examined muscles exhibited lower activation only during a small portion of the sway cycle, while the overall activation pattern remained similar across both velocities (29), indicating no relevant effects of visual target motion frequency.

In conclusion, our results show a decreased similarity in activation patterns within the synergistic triceps surae and quadriceps femoris muscle pairs on the soft surface indicating an increased flexibility of neuromotor control in the unstable condition. The lower CS in the activation patterns of triceps surae muscle pairs compared to the quadriceps femoris muscle pairs suggests a higher diversity in activation, which may increase the flexibility of neuromotor control to meet specific joint stabilization challenges during the investigated tracking task. These insights into postural control mechanisms offer potential applications for balance training and rehabilitation, where enhancing flexible motor strategies might improve stability in variable challenging environments. The critical role of the human plantar flexor muscles in controlling body stability under demanding locomotor conditions has been widely reported in the past (44, 47, 48). For example, selective EMG activity enhancement of the GM muscle has been found during drop-like perturbations (44, 48). It may be possible to influence the flexibility and diversity of synergistic muscles, in particular the triceps surae muscles, which are very important for balance performance (49, 50), by increasing the balance task complexity and difficulty using unstable conditions.

## Data Availability

The raw data supporting the conclusions of this article will be made available by the authors, without undue reservation.

## References

[1] Bizzi E Cheung VCK d’Avella A Saltiel P Tresch M. Combining modules for movement. Brain Res Rev. (2008) 57:125–33. 10.1016/j.brainresrev.2007.08.004 18029291 PMC4295773

[2] Dominici N Ivanenko YP Cappellini G D’Avella A Mondì V Cicchese M Locomotor primitives in newborn babies and their development. Science. (2011) 334:997–9. 10.1126/science.1210617 22096202

[3] Clark DJ Ting LH Zajac FE Neptune RR Kautz SA. Merging of healthy motor modules predicts reduced locomotor performance and muscle coordination complexity post-stroke. J Neurophysiol. (2010) 103:844–57. 10.1152/jn.00825.2009 20007501 PMC2822696

[4] Brüll L Hezel N Arampatzis A Schwenk M. Comparing the effects of two perturbation-based balance training paradigms in fall-prone older adults: a randomized controlled trial. Gerontology. (2023) 69:910–22. 10.1159/000530167 36921581

[5] Xu D Zhou H Quan W Ma X Chon TE Fernandez J New insights optimize landing strategies to reduce lower limb injury risk. Cyborg Bionic Syst. (2024) 5:0126. 10.34133/cbsystems.0126 38778877 PMC11109754

[6] Oliveria SA Gizzi CL Kersting UG Farina D. Modular organization of balance control following perturbations during walking. J Neurophysiol. (2012) 108:1895–906. 10.1152/jn.00217.2012.-Balance 22773783

[7] Munoz-Martel V Santuz A Ekizos A Arampatzis A. Neuromuscular organisation and robustness of postural control in the presence of perturbations. Sci Rep. (2019) 9:12273. 10.1038/s41598-019-47613-7 31439926 PMC6706387

[8] Cappellini G Ivanenko YP Poppele RE Lacquaniti F. Motor patterns in human walking and running. J Neurophysiol. (2006) 95:3426–37. 10.1152/jn.00081.2006 16554517

[9] Ting LH Chiel HJ Trumbower RD Allen JL McKay JL Hackney ME Neuromechanical principles underlying movement modularity and their implications for rehabilitation. Neuron. (2015) 86:38–54. 10.1016/j.neuron.2015.02.042 25856485 PMC4392340

[10] Janshen L Santuz A Ekizos A Arampatzis A. Fuzziness of muscle synergies in patients with multiple sclerosis indicates increased robustness of motor control during walking. Sci Rep. (2020) 10:7249. 10.1038/s41598-020-63788-w 32350313 PMC7190675

[11] Santuz A Ekizos A Kunimasa Y Kijima K Ishikawa M Arampatzis A. Lower complexity of motor primitives ensures robust control of high-speed human locomotion. Heliyon. (2020b) 6:e05377. 10.1016/j.heliyon.2020.e05377 33163662 PMC7610320

[12] Munoz-Martel V Santuz A Bohm S Arampatzis A. Proactive modulation in the spatiotemporal structure of muscle synergies minimizes reactive responses in perturbed landings. Front Bioeng Biotechnol. (2021b) 9:761766. 10.3389/fbioe.2021.761766 34976964 PMC8716881

[13] Xu D Zhou H Quan W Gusztav F Baker JS Gu Y. Adaptive neuro-fuzzy inference system model driven by the non-negative matrix factorization-extracted muscle synergy patterns to estimate lower limb joint movements. Comput Methods Programs Biomed. (2023) 242:107848. 10.1016/j.cmpb.2023.107848 37863010

[14] Martino G Ivanenko YP Serrao M Ranavolo A Draicchio F Conte C Locomotor patterns in cerebellar ataxia. J Neurophysiol. (2014) 112:2810–21. 10.1152/jn.00275.2014.-Several 25185815

[15] Martino G Ivanenko YP D’avella A Serrao M Ranavolo A Draicchio F Neuromuscular adjustments of gait associated with unstable conditions. J Neuro-physiol. (2015) 114:2867–82. 10.1152/jn.00029.2015.-A PMC4737426 26378199

[16] Santuz A Brüll L Ekizos A Schroll A Eckardt N Kibele A Neuromotor dynamics of human locomotion in challenging settings. iScience. (2020a) 23:100796. 10.1016/j.isci.2019.100796 31962235 PMC6971393

[17] Santuz A Ekizos A Eckardt N Kibele A Arampatzis A. Challenging human locomotion: stability and modular organisation in unsteady conditions. Sci Rep. (2018) 8:2740. 10.1038/s41598-018-21018-4 29426876 PMC5807318

[18] Munoz-Martel V Santuz A Bohm S Arampatzis A. Neuromechanics of dynamic balance tasks in the presence of perturbations. Front Hum Neurosci. (2021a) 14:560630. 10.3389/fnhum.2020.560630 33584219 PMC7874030

[19] Mademli L Mavridi D Bohm S Patikas DA Santuz A Arampatzis A. Standing on unstable surface challenges postural control of tracking tasks and modulates neuromuscular adjustments specific to task complexity. Sci Rep. (2021) 11:6122. 10.1038/s41598-021-84899-y 33731729 PMC7969732

[20] Héroux ME Dakin CJ Luu BL Inglis JT Blouin J-S. Absence of lateral gastrocnemius activity and differential motor unit behavior in soleus and medial gastrocnemius during standing balance. J Appl Physiol. (2014) 116:140–8. 10.1152/japplphysiol.00906.2013 24311748 PMC3921363

[21] Hug F Del Vecchio A Avrillon S Farina D Tucker K. Muscles from the same muscle group do not necessarily share common drive: evidence from the human triceps surae. J Appl Physiol. (2021) 130:342–54. 10.1152/JAPPLPHYSIOL.00635.2020 33242301

[22] Rossato J Tucker K Avrillon S Lacourpaille L Holobar A Hug F. Less common synaptic input between muscles from the same group allows for more flexible coordination strategies during a fatiguing task. J Neurophysiol. (2022) 127:421–33. 10.1152/jn.00453.2021 35020505

[23] Sheehan FT Borotikar BS Behnam AJ Alter KE. Alterations in *in vivo* knee joint kinematics following a femoral nerve branch block of the vastus medialis: implications for patellofemoral pain syndrome. Clin Biomech. (2012) 27:525–31. 10.1016/j.clinbiomech.2011.12.012 PMC3328589 22244738

[24] Alessandro C Barroso FO Prashara A Tentler DP Yeh H-Y Tresch MC Coordination amongst quadriceps muscles suggests neural regulation of internal joint stresses, not simplification of task performance. PNAS. (2020) 117:8135–42. 10.1073/pnas.1916578117/-/DCSupplemental 32205442 PMC7149390

[25] Lai AKM Lichtwark GA Schache AG Pandy MG. Differences in *in vivo* muscle fascicle and tendinous tissue behavior between the ankle plantarflexors during running. Scand J Med Sci Sports. (2018) 28:1828–36. 10.1111/sms.13089 29603434

[26] Hamard R Aeles J Kelp NY Feigean R Hug F Dick TJM. Does different activation between the medial and the lateral gastrocnemius during walking translate into different fascicle behavior? J Exp Biol. (2021) 224:jeb242626. 10.1242/jeb.242626 34096594

[27] Mersmann F Bohm S Schroll A Marzilger R Arampatzis A. Athletic training affects the uniformity of muscle and tendon adaptation during adolescence. J Appl Physiol. (2016) 121:893–9. 10.1152/japplphysiol.00493.2016.-With 27586836

[28] Brüll L Santuz A Mersmann F Bohm S Schwenk M Arampatzis A. Spatiotemporal modulation of a common set of muscle synergies during unpredictable and predictable gait perturbations in older adults. J Exp Biol. (2024) 227:jeb247271. 10.1242/jeb.247271 38506185 PMC11058090

[29] Patikas DA Papavasileiou A Ekizos A Hatzitaki V Arampatzis A. Swaying slower reduces the destabilizing effects of a compliant surface on voluntary sway dynamics. PLoS One. (2019) 14:e0226263. 10.1371/journal.pone.0226263 31826026 PMC6905565

[30] Sotirakis H Kyvelidou A Mademli L Stergiou N Hatzitaki V. Aging affects postural tracking of complex visual motion cues. Exp Brain Res. (2016) 234:2529–40. 10.1007/s00221-016-4657-x 27126061 PMC5253232

[31] Sotirakis H Hatzitaki V Munoz-Martel V Mademli L Arampatzis A. Center of pressure feedback modulates the entrainment of voluntary sway to the motion of a visual target. Appl Sci. (2019) 9:3952. 10.3390/app9193952

[32] Jacqmin-Gadda H Sibillot S Proust C Molina JM Thiébaut R. Robustness of the linear mixed model to misspecified error distribution. Comput Stat Data Anal. (2007) 51:5142–54. 10.1016/j.csda.2006.05.021

[33] Daley MA Biewener AA. Running Over Rough Terrain Reveals Limb control for Intrinsic Stability (2006). Available online at: www.pnas.orgcgidoi10.1073pnas.0601473103 10.1073/pnas.0601473103 PMC1622881 17032779

[34] Biewener AA Daley MA. Unsteady locomotion: integrating muscle function with whole body dynamics and neuromuscular control. J Exp Biol. (2007) 210:2949–60. 10.1242/jeb.005801 17704070 PMC2651961

[35] Lee SSM Piazza SJ. Inversion-eversion moment arms of gastrocnemius and tibialis anterior measured *in vivo*. J Biomech. (2008) 41:3366–70. 10.1016/j.jbiomech.2008.09.029 19019375

[36] Winter DA. Human balance and posture control during standing and walking. Gait Posture. (1995) 3:193–214. 10.1016/0966-6362(96)82849-9

[37] Loram ID Lakie M. Direct measurement of human ankle stiffness during quiet standing: the intrinsic mechanical stiffness is insufficient for stability. J Physiol. (2002) 545:1041–53. 10.1113/jphysiol.2002.025049 12482906 PMC2290720

[38] Loram ID Maganaris CN Lakie M. Paradoxical muscle movement during postural control. Med Sci Sports Exerc. (2009) 41:198–204. 10.1249/MSS.0b013e318183c0ed 19092688

[39] Paillard T. Relationship between muscle function, muscle typology and postural performance according to different postural conditions in young and older adults. Front Physiol. (2017) 8:585. 10.3389/fphys.2017.00585 28861000 PMC5559497

[40] Albracht K Arampatzis A Baltzopoulos V. Assessment of muscle volume and physiological cross-sectional area of the human triceps surae muscle *in vivo*. J Biomech. (2008) 41:2211–8. 10.1016/j.jbiomech.2008.04.020 18555257

[41] Winkler T Mersmann F Von Roth P Dietrich R Bierbaum S Arampatzis A. Development of a non-invasive methodology for the assessment of muscle fibre composition. Front Physiol. (2019) 10:174. 10.3389/fphys.2019.00174 30914961 PMC6421337

[42] Schenau GJVI. From rotation to translation: constraints on multi-joint movements and the unique action of bi-articular muscles. Hum Mov Sci. (1989) 8:301–37. 10.1016/0167-9457(89)90037-7

[43] Arampatzis A Kharazi M Theodorakis C Mersmann F Bohm S. Biarticular mechanisms of the gastrocnemii muscles enhance ankle mechanical power and work during running. R Soc Open Sci. (2023) 10:230007. 10.1098/rsos.230007 37650058 PMC10465202

[44] Nakazawa K Kawashima N Akai M Yano H. On the reflex coactivation of ankle flexor and extensor muscles induced by a sudden drop of support surface during walking in humans. J Appl Physiol. (2004) 96:604–11. 10.1152/japplphysiol.00670.2003.-Recent 14527965

[45] McDonagh MJN Duncan A. Interaction of pre-programmed control and natural stretch reflexes in human landing movements. J Physiol. (2002) 544:985–94. 10.1113/jphysiol.2002.024844 12411541 PMC2290625

[46] Patel M Fransson PA Lush D Gomez S. The effect of foam surface properties on postural stability assessment while standing. Gait Posture. (2008) 28:649–56. 10.1016/j.gaitpost.2008.04.018 18602829

[47] Dick TJM Clemente CJ Punith LK Sawicki GS. Series elasticity facilitates safe plantar flexor muscle-tendon shock absorption during perturbed human hopping. Proc R Soc B. (2021) 288:20210201. 10.1098/rspb.2021.0201 33726594 PMC8059679

[48] Bunz EK Haeufle DFB Remy CD Schmitt S. Bioinspired preactivation reflex increases robustness of walking on rough terrain. Sci Rep. (2023) 13:13219. 10.1038/s41598-023-39364-3 37580375 PMC10425464

[49] Dick TJM Punith LK Sawicki GS. Humans falling in holes: adaptations in lower-limb joint mechanics in response to a rapid change in substrate height during human hopping. J R Soc Interface. (2019) 16:20190292. 10.1098/rsif.2019.0292 31575349 PMC6833322

[50] Golyski PR Sawicki GS. Which lower limb joints compensate for destabilizing energy during walking in humans? J R Soc Interface. (2022) 19:20220024. 10.1098/rsif.2022.0024 35642426 PMC9156907

